# Stress-Dependent Changes in the CacyBP/SIP Interacting Protein S100A6 in the Mouse Brain

**DOI:** 10.1371/journal.pone.0169760

**Published:** 2017-01-09

**Authors:** Katarzyna Bartkowska, Izabela Swiatek, Agata Aniszewska, Ewelina Jurewicz, Kris Turlejski, Anna Filipek, Rouzanna L. Djavadian

**Affiliations:** 1 Nencki Institute of Experimental Biology Polish Academy of Sciences, Warsaw, Poland; 2 Cardinal Stefan Wyszynski University in Warsaw, Poland; Technion Israel Institute of Technology, ISRAEL

## Abstract

The CacyBP/SIP target S100A6 is widely present in the nervous system, and its up-regulation is associated with certain neurodegenerative diseases. Here, we examined the involvement of S100A6 protein in stress responses in mice. Using Western blotting, we observed a marked change in brainstem structures, whereby stressed mice showed approximately one-third the protein level produced in the control group. A decreased level of S100A6 protein in stressed animals was also detected in the olfactory bulb and the cerebellum and stress-related structures such as the hippocampus and the hypothalamus. Additionally, using immunohistochemistry, high levels of S100A6 expression were observed in astrocytes localized in the border zones of all brain ventricles, tanycytes of the ventro-lateral walls of the hypothalamus, including the arcuate nucleus (ARH) and low levels of this protein were in neurons of the olfactory bulb, the hippocampus, the thalamus, the cerebral cortex, the brainstem and the cerebellum. Although S100A6-expressing cells in all these brain structures did not change their phenotype in response to stress, the intensity of immunofluorescent labeling in all studied structures was lower in stressed mice than in control animals. For example, in the ARH, where extremely strong immunostaining was observed, the number of immunolabeled fibers was decreased by approximately half in the stressed group compared with the controls. Although these results are descriptive and do not give clue about functional role of S100A6 in stress, they indicate that the level of S100A6 decreases in several brain structures in response to chronic mild stress, suggesting that this protein may modify stress responses.

## Introduction

S100A6 (calcyclin) belongs to the family of low-molecular-weight calcium-binding proteins [1]. The protein was originally isolated from Ehrlich ascites tumor cells and was subsequently found in other mammalian tissues, including brain. In the brain, S100A6 protein was first described in neurons of the hippocampus, the cerebellum and the brain stem [2]. However, expression of S100A6 is not restricted to neurons of these brain regions; S100A6 is observed in a wide variety of brain structures, with high expression in neurons and in astrocytes and ependymal cells of the brain [3].

Elevated expression of S100A6 is associated with both normal and pathological aging processes [4, 5]. In the young mouse brain, astrocytes containing S100A6 are located around the ventricles and in the white matter, mainly in the corpus callosum, external and internal capsules, the fimbria of the hippocampus and the white matter of the spinal cord. It has been suggested that S100A6 is involved in the regulation of normal aging. During physiological aging, S100A6 expression becomes visible and strong in astrocytes of the hippocampus, whereas it is absent from those cells in young animals [5]. In the human aged brain, the level of S100A6 in glial-like cells of the occipital cortex is also elevated [4].

In addition, the high level of S100A6 is assumed to play an important role in certain neurodegenerative diseases. In the case of amyotrophic lateral sclerosis, which is characterized by the degeneration of motoneurons that control muscle movement, S100A6 is selectively up-regulated within astrocytes surrounding the neurodegenerative lesions [6, 7]. One of the possible mechanisms by which S100A6 may lead to motoneuron death is through the depletion of zinc (Zn). In normal physiological conditions, S100A6 in astrocytes can bind Zn^2+^ and Ca^2+^, but in transgenic amyotrophic lateral sclerosis mice model, where astrogliosis occurs, S100A6 prevents its binding with Zn^2+^, causing Zn deficiency due to decreased zinc affinity [8, 9].

In dementia common in the elderly population, such as Alzheimer’s disease, astrocytic S100A6 is homogeneously up-regulated within the white matter and in the neocortex, almost all S100A6 immunoreactivity is concentrated in astrocytes surrounding the amyloid beta deposits of senile plaques [10].

S100A6 interacts with many protein ligands; among them is CacyBP/SIP, which interacts with S100A6 at physiological concentrations of Ca^2+^ [11]. Interestingly, it was found that S100A6 might influence the phosphatase activity of CacyBP/SIP towards tau, a protein involved in Alzheimer’s disease pathology [12]. Generally, patients with Alzheimer’s disease have high levels of stress hormones, which may indicate a change in the regulation of the hypothalamic-pituitary-adrenal (HPA) axis. As a result, they have a reduced activity of the immune system. We therefore investigated whether the level of S100A6 can be modulated by stress.

The aim of the current study was to characterize the stress-dependent effects on S100A6 in various brain structures and to identify the phenotype of S100A6-expressing cell populations in response to chronic unpredictable stress.

## Materials and Methods

### Animals

Male C57BL6/J mice at 4–5 months old (n = 19) were used in this study. They were kept at 23°C and on a 12-hour light-dark cycle in the animal house of the Nencki Institute of Experimental Biology PAS. All efforts were made to minimize the number of animals used and the amount of stress placed upon them. Experimental procedures complied with the Polish Law on Experimentation on Animals that implements the European Council Directive of 24 November 1986 (86/609/EEC) and the NIH Guide for the Care and Use of Laboratory Animals. The experiments were approved and controlled by the First Local Ethics Committee in Warsaw (Resolution Nr 622/2014).

### Induction of stress

Mice were divided into two groups: control and stressed. On the first day, the mice in the stressed group were subjected to the forced swimming task. Animals were placed into a cylinder of 12 cm in diameter and 20 cm high, filled with water at 17–18°C to a height of 15 cm. After 10 min of forced swimming, mice were dried with a paper towel and returned to their home cages. On the second day, mice were presented with a stressor smell in the form of rat feces placed in a mouse home cage overnight. The next day, the mice were kept in a room in which 24 h intensive light (400 lumens) was switched on. On the fourth day, the mice were again subjected to the forced swimming task. Twenty-four hours later, half of the mice from each group were sacrificed by decapitation after intraperitoneal injection with a lethal dose of pentobarbital (100 mg/kg) for preparation of protein lysates, and the other half of the mice were perfused as described below.

### Immunohistochemistry and histology

Five mice from each of the control and stressed groups were used for immunohistochemistry. Each mouse was injected intraperitoneally with a lethal dose of pentobarbital (100 mg/kg) and then was perfused transcardially with 4% paraformaldehyde in 0.1 M phosphate buffer. The brains were removed, postfixed overnight in the same 4% paraformaldehyde solution, then soaked in 10%, 20% and 30% sucrose (24 h each) and frozen at -70°C prior to sectioning. The brains were then cut into 40-μm sections on a cryostat. Ten series of sections were collected.

Sections were rinsed in PBS buffer (pH 7.4), transferred to 3% H_2_O_2_ with 10% methanol for 30 min, and then rinsed for 30 min with PBS buffer containing 0.1% Triton X-100 (Sigma-Aldrich, St. Louis, MO). To block endogenous peroxidase activity, sections were incubated in a solution containing 10% normal goat serum (Millipore, Temecula, CA) and 5% bovine serum albumin (Sigma-Aldrich, St. Louis, MO). They were then incubated overnight with the primary antibody, rabbit anti-S100A6 (made in house) diluted 1:40, in PBS. After rinsing, sections were incubated in secondary goat anti-rabbit antibody conjugated with Alexa Fluor 568 in PBS (1:500, Invitrogen, Carlsbad, CA), rinsed again and blocked with 10% normal donkey serum (Sigma Aldrich, St. Louis, MO) or normal chicken serum (Abcam, Cambridge, UK) and 5% bovine serum albumin in PBS. Next, sections were incubated overnight in PBS with a primary antibody targeting one of the following proteins: NeuN (1:400, Millipore, Temecula, CA), GFAP (1:500, Sigma-Aldrich, St. Louis, MO), olig2 (1:100, Millipore, Temecula, CA), SOX2 (1:200, Santa Cruz Biotechnology, Dallas, TX), MBP (1:100, Santa Cruz Biotechnology, Dallas, TX), MAP2 (1:200, Abcam, Cambridge, UK), EAAT1 (1:100, Santa Cruz Biotechnology, Dallas, TX) or VIM (1:300, Sigma-Aldrich, St. Louis, MO. Labeling was visualized with secondary antibodies conjugated with Alexa Fluor 488 (donkey anti-chicken, 1:500, Abcam, Cambridge, UK; chicken anti-goat, 1:400, Invitrogen, Carlsbad, CA and chicken anti-mouse, 1:600, Invitrogen, Carlsbad, CA). All sections were stained with 4′,6-diamidino-2-phenylindole (DAPI, Sigma-Aldrich, St. Louis, MO). Sections were mounted using Mounting Medium (Sigma-Aldrich, St. Louis, MO).

To identify brain structures, one series of sections was counterstained with Nissl stain.

### Western blot analyses

The following brain structures were chosen for measuring the level of S100A6: the olfactory bulb and the subventricular zone of the lateral ventricle (SVZ) were selected as structures that are involved in adult neurogenesis; the hippocampus, the hypothalamus, the rostral part of the cerebral cortex and the brainstem were studied in relation to stress, and the cerebellum was chosen as a structure of reference that is not supposed to be involved in stress responses. Fresh samples of various brain regions were homogenized using a Polytron homogenizer at 6000 rpm in a solution containing 20 mM Tris pH 7.5, 0.5 mM PMSF, 1 mM EDTA, 1 mM DTT (Sigma-Aldrich, St. Louis, MO), cOmplete, EDTA-free Protease Inhibitor Cocktail (Roche/ Sigma-Aldrich, St. Louis, MO) and detergents 1% NP-40 (Sigma-Aldrich, St. Louis, MO) and 0.1% SDS (Sigma-Aldrich, St. Louis, MO). The homogenates were then centrifuged at 13 900 x g for 45 min and 0.5 M NaCl (Avantor Performance Materials Poland S.A, Gliwice, Poland) and 3 mM CaCl_2_ (Avantor Performance Materials Poland S.A, Gliwice, Poland) was added to the supernatant and protein concentration was estimated by the Bradford procedure with BSA as a standard (Bradford Protein Assay, BioRad Laboratories, Hercules, CA).

Proteins (50 μg) were separated by SDS-PAGE (10%) using the method of Laemmli. After transfer onto nitrocellulose, proteins were identified by immunoblotting using the following primary antibodies as appropriate: rabbit anti-S100A6 polyclonal antibody (1:250, made in house) or mouse anti-GAPDH monoclonal antibody (1:10000, Millipore, Temecula, CA). Blots were washed with TBS-T buffer (50 mM Tris pH 7.5, 200 mM NaCl, 0.05% Tween 20) and then allowed to react with secondary antibodies, either goat anti-rabbit IgG (1:10000, BioRad Laboratories, Hercules, CA) or goat anti-mouse IgG (1:15000, Millipore, Temecula, CA). After 3 washes with TBS-T and 2 washes with TBS (50 mM Tris pH 7.5, 200 mM NaCl), blots were developed with the ECL chemiluminescence kit (Advansta, Henlo Park, CA).

### Data analysis and statistics

Western blots were scanned with a G:Box (Syngene, Cambridge, UK), and intensities of protein bands were quantified using Syngene GeneSys software. For quantification of protein expression, the values obtained for S100A6 bands were normalized to those of GAPDH. For statistical calculations, a two-tailed paired Student's *t*-test was performed to compare values between the two groups, control and stressed. Normalized values from 3 independent experiments were used for statistical analysis. The results are shown as the mean ± S.E. To compare more than two structures, one-way analysis of variance (ANOVA) followed by a post-hoc multiple pairwise comparisons test (Tukey’s test) was used.

The immunostained sections were examined and photographed using an Eclipse 90i microscope (Nikon) equipped with a digital camera. Neurolucida software was used for performing brain mapping.

Colocalization of the three fluorescent labels was determined using a confocal laser microscope (Zeiss). Slide-mounted brain sections were viewed and scanned with 488 nm, 556 nm and DAPI laser lines for detection of the corresponding Alexa fluorophores. To quantify the percentage of double-labeled cells, at least 100 S100A6-positive cells were analyzed from each selected brain structure from each animal. The numbers of cells showing colocalization of S100A6 and NeuN, of S100A6 and GFAP, and of S100A6 and SOX2 were then calculated.

To estimate the intensity of immunofluorescence staining, ImageJ software was used. The intensity of immunofluorescne staining corrected against the background signal and the data were presented as the ratio staining/background.

## Results

### Influence of stress on S100A6 protein levels in the brain

**Fig 1:**
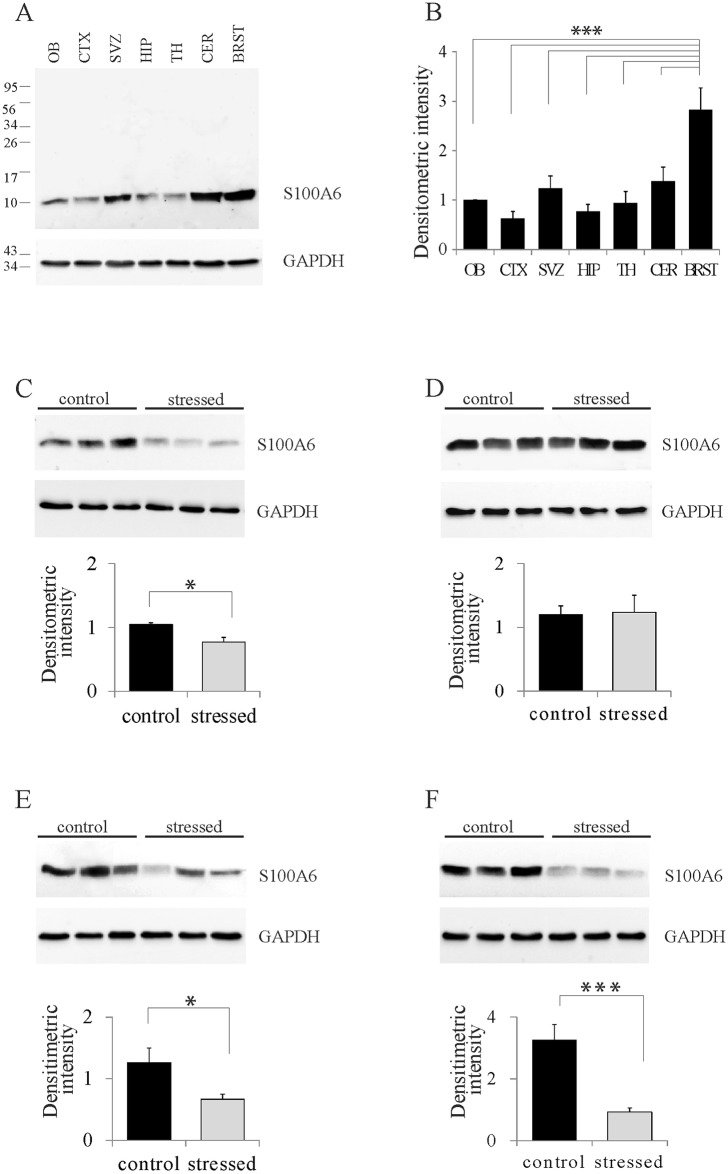
Results of western blots showing the protein levels in different brain structures. (A) Western blot showing S100A6 and the corresponding loading control, GAPDH protein, from various brain structures of control mice. (B) Graphs showing a densitometric analysis of data and a quantification of protein levels normalized to GAPDH protein. Each bar corresponds to one brain structure (OB—the olfactory bulbs, CTX—the cerebral cortex, SVZ—the subventricular zone of the lateral ventricle, HP—the hippocampus, TH—the thalamus and the hypothalamus, CER—the cerebellum, BRST—the brainstem structures). (C–F) Western blots and graphs showing densitometric analysis of data from the OB, SVZ, CER, and BRST respectively, in control and stressed mice. Mean values ±SE from n = 4 per group. * p<0.05, ***p<0.0001.

To test whether stress alters expression of S100A6 in various brain regions, protein lysates were obtained from selected brain structures, including those related to stress, and then analyzed using Western blotting. In control animals, a specific band at approximately 10 kDa was detected; this value is consistent with the molecular mass of the S100A6 monomer (Fig 1A). We found that the intensity of the band varied among brain structures. The highest level of S100A6 expression was detected in brainstem structures (Fig 1B). In the subventricular zone of the lateral ventricle (SVZ) and the cerebellum, the level of S100A6 was high and in the other brain structures analyzed, the amount of S100A6 protein was moderate (Fig 1A and 1B). One-way ANOVA analysis revealed that differences in the S100A6 protein content between structures were significant (p<0.005).

Next, we compared the level of S100A6 in those selected brain structures between control and stressed animals. The level of S100A6 was decreased in response to stress compared with the control group. The most dramatic changes were observed in brainstem structures, where its level in stressed animals was approximately one-third that in control animals (Fig 1F). Significant differences in the level of the S100A6 protein were also found in the olfactory bulb (Fig 1C), the cerebellum (Fig 1E), the hippocampus and the hypothalamus.

### Identification of the cell type expressing S100A6 in stress responses

**Fig 2:**
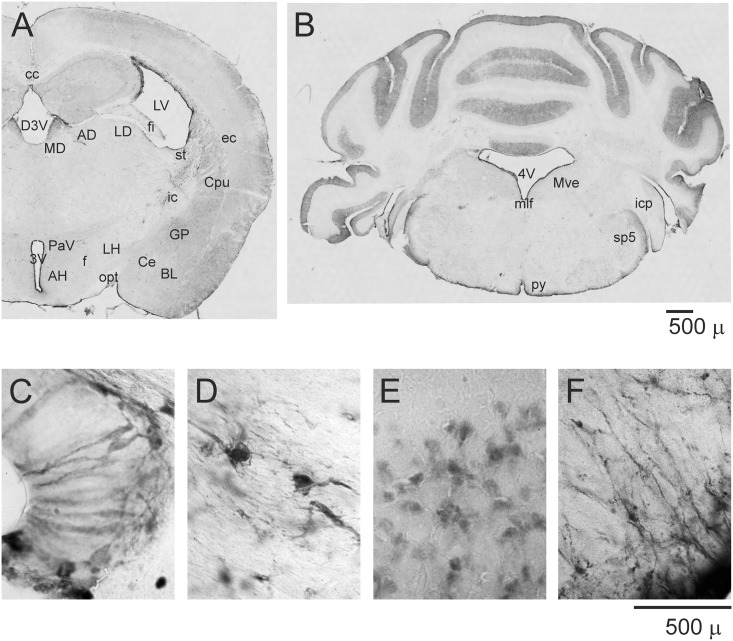
S100A6-immunoreactivity in mouse brain structures. (A, B) Coronal sections from anterior and caudal regions of the brain. (C-F) High-magnification images showing S100A6 immunolabeling in the zone close to the dorsal 3rd ventricle (D3V), the corpus callosum (cc), the cerebellum and the pyramidal tract (py). MD—medial dorsal nucleus, AD—anterodorsal nucleus, LD—lateral dorsal nucleus, LV—lateral ventricle, Cpu—caudate-putamen, GP—globus pallidus, Ce—central amygdaloid nucleus, BL—basal amygdaloid nucleus, LH—lateral hypothalamic area, PaV—paraventricular hypothalamic nucleus, AH—anterior hypothalamus, Mve—medial vestibular nucleus, 3V—3rd ventricle, 4V—4th ventricle, ec—external capsule, ic—internal capsule, opt—optic tract, fi—fimbria of hippocampus, st—stria terminalis, sp5- spinal trigeminal tract, icp—inferior cerebellar peduncle, mlf—medial longitudinal fasciculus.

To address whether the S100A6 protein is located in specific cell populations in the brain in response to stress, we examined immunostained brain sections from control and stressed mice. S100A6 is an abundant protein present in many structures, and its distribution in mouse brain is similar to that in rat [3]. We found that S100A6-immunopositive cells were stained with varying intensity in different brain regions (Fig 2). Strong immunoreactivity was observed in all zones close to the brain ventricles, the cerebral cortex, the hippocampus, the arcuate nucleus, the amygdala, the cerebellum, and some brainstem structures, as well as major fiber tracts such as the corpus callosum, anterior commissure, fimbria of the hippocampus, external capsule and internal capsule.

**Fig 3:**
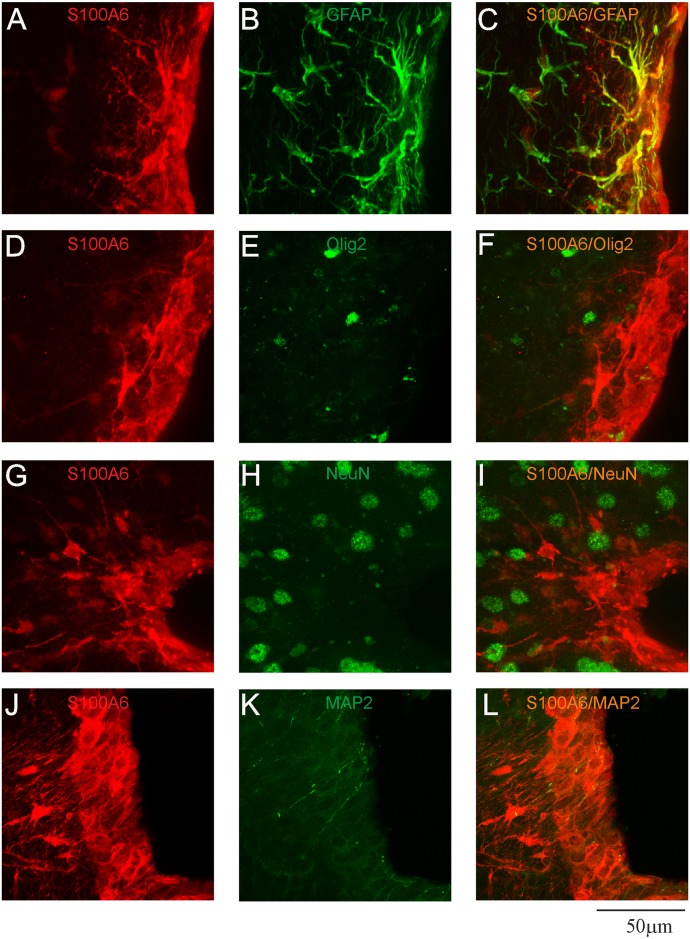
Confocal images presenting the phenotypes of S100A6-immunopositive cells in the SVZ. (A, D, G, J) confocal analysis with anti-S100A6 antibody (red) and (B) anti-GFAP (astrocytic marker, green), (E) anti-Olig2 (oligodendrocytic marker, green), (H) anti-NeuN (neuronal marker, green), (K) anti-MAP2 (dendritic marker, green); (C, F, I, L) merged images.

To determine the phenotype of cells expressing S100A6, double- or triple-labeling immunohistochemistry was performed (Fig 3). In all zones close to the brain ventricles, including the cerebral aqueduct, the majority of cells stained for GFAP, which is a marker of astrocytes, were S100A6-immunoreactive (Fig 3A–3C). Another type of glial cell, oligodendrocytes, also contained S100A6 (Fig 3D–3F), however, no neurons were labeled with anti-S100A6 antibody (Fig 3G–3I).

**Fig 4:**
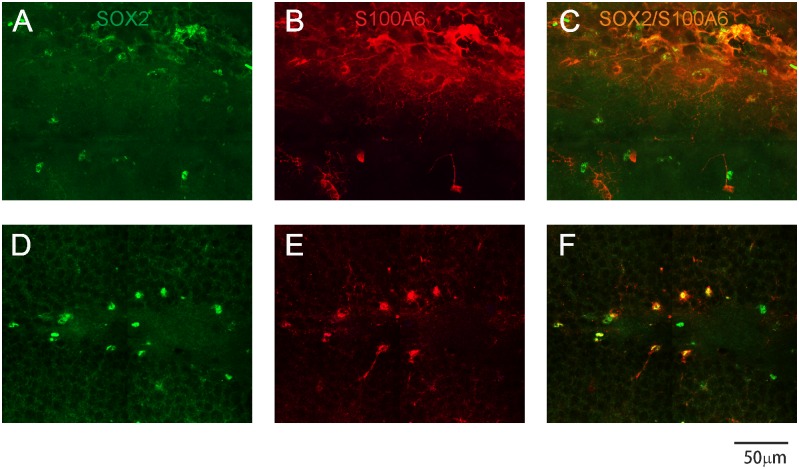
Confocal images of the SVZ and the DG. Sections were stained with antibodies specific for S100A6 (red) and SOX2 (progenitor cell marker, green). (C, F) merged images. Note that a majority of S100A6-labeled cells colocalize with SOX2 in the DG, indicating that S100A6 is expressed in progenitor cells of the DG (F), while weak colocalization between S100A6 and SOX2 in the SVZ (C) is seen.

Although the majority of S100A6-positive cells in the SVZ of the lateral ventricles were astrocytes, we were not able to count these cells because astrocytes’ somas were encircled by dense tangled fibers, making them invisible. Therefore, we decided to use an antibody against SOX2, a marker for cell proliferation that is also known as SRY (sex determining region Y)-box 2, to stain nuclei. Based on data by Yamada and Jinno (2014) S100A6 has been considered a specific marker of proliferating cells in the adult hippocampus. We found that 86% of SOX2-positive cells were located in one of the germinal zones in the dentate gyrus (DG) of adult mouse brain (Fig 4D–4F), which was consistent with previous data (Yamada and Jinno, 2012). However, in the SVZ of the lateral ventricle, SOX2 expression was low, and only a minority of SOX2-expressing cells (approximately 30%) stained for S100A6 (Fig 4A–4C). Furthermore, to quantify these results, we analyzed the mean density of the fluorescence signal. In comparison with control animals, stressed mice showed a decrease in the intensity of immunofluorescent labeling.

**Fig 5:**
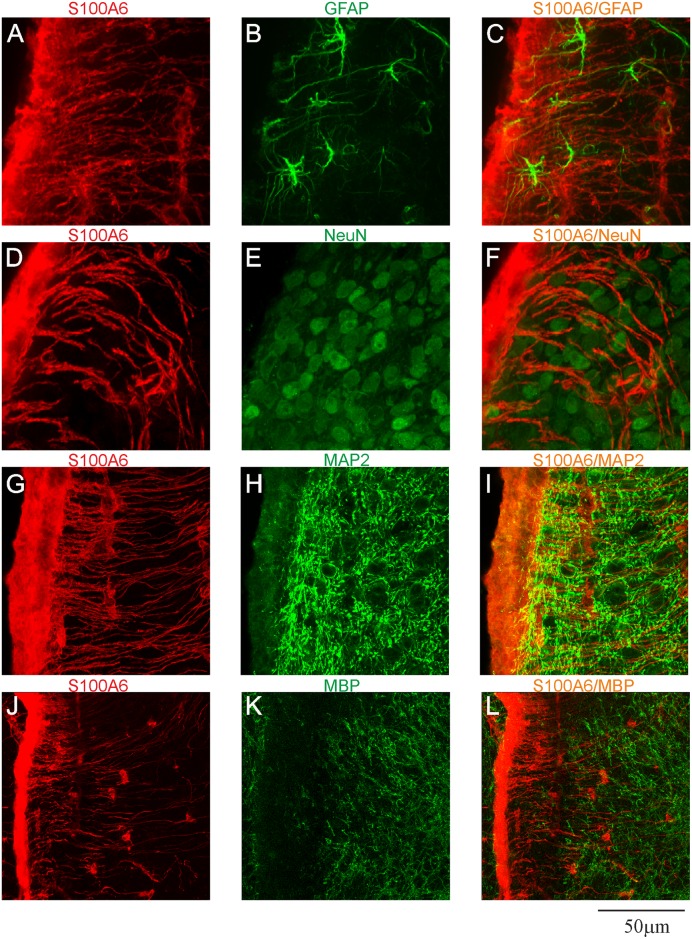
Confocal images presenting the phenotypes of S100A6-immunopositive cells in the ARH. (A, D, G, J) confocal analysis with anti-S100A6 antibody (red) and (B) anti-GFAP (astrocytic marker, green), (E) anti-NeuN (neuronal marker, green), (H) anti-MBP (myelin basic protein, green), (K) anti-MAP2 (dendritic marker, green). (C, F, I, L) merged images.

We next investigated the influence of stress on the pattern of S100A6 expression in the arcuate nucleus of the hypothalamus (ARH), which was very strongly stained by the antibody against S100A6 (Fig 5A, 5D, 5G and 5J). The ARH is located adjacent to the third ventricle and consists of groups of projection neurons. Cells and processes within this nucleus were intensely labeled with antibody against S100A6. Analyzing the colocalization of GFAP and S100A6 in confocal microscope images, we did not observe any astrocytes in the ARH that expressed S100A6 (Fig 5A–5C). Intense NeuN staining was observed in the nuclei of arcuate neurons (Fig 5E), whereas S100A6 labeling was present in the processes of cells (Fig 5F). The pattern of immunostaining was similar to that in radial glia, but using an antibody against GLAST, which is a marker for radial glia, we were unable to find colocalization with S100A6 in the same fibers. To define the cell type to which these S100A6-labeled fibers belonged, we also used the dendritic marker, microtubule-associated protein 2 (MAP2), and the axonal marker, myelin basic protein (MBP). These analyses also failed to detect cell processes that simultaneously stained for both S100A6 and MAP2 (Fig 5G–5I) or S100A6 and MBP (Fig 5J–5L). Fig 5J–5L illustrates that a substantial number of S100A6-positive fibers were not myelinated axons.

**Fig 6:**
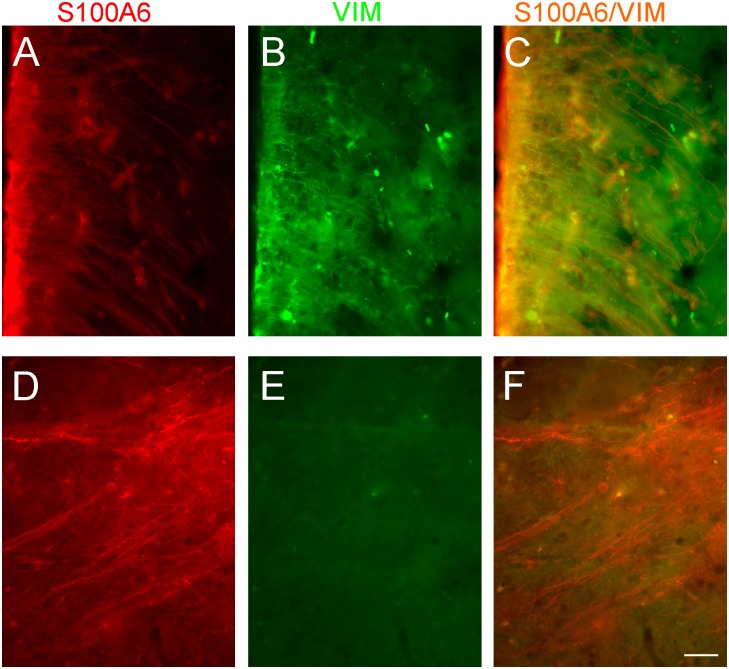
Confocal images of brain sections immunostained with S100A6 and vimentin antibodies. (A, B) the coronal section of the ARH stained for S100A6 (red) and vimentin (green). (D, E) the coronal section of the inferior cerebellar peduncle labeled with S100A6 (red) and vimentin (green). (C, F) merged images. Scale bar equals 50 μ.

Immunolabeled S100A6 cells in the ARH were localized mainly in the ventro-lateral walls of the third ventricle and seemed to be tanycytes. To visualize tanycytes we performed immunohistochemistry using vimentin antibody. We observed that a majority of cells in the ARH stained for vimentin, simultaneously expressed S100A6 (Fig 6A–6C), indicating that they were hypothalamic tanycytes. In other brain regions containing heavy S100A6-immunoreactive fibers, tanycytes were not present. For example, in the inferior cerebellar peduncle S100A stained fibers were not expressed vimentin as shown in the Fig 6D–6F.

**Fig 7:**
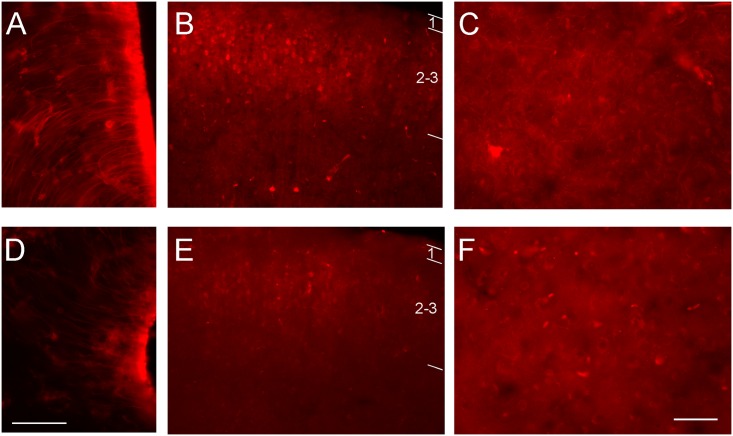
Representative immunofluorescence images for S100A6 in some brain structures related to stress responses. Immunostained images for S100A6 in the ARH, cerebral cortex and amygdala of control (A-C) and stressed mice (D-F). Scale bars equal 50 μ.

The intensity of S100A6 stained fibers in the ARH in stressed mice was lower than in the controls (3,7 vs 5,2 respectively, p<0.04) animals (Fig 7A and 7D). Moreover, the number of S100A6-stained fibers was decreased in the stressed group relative to the controls. The intensity of S100A6 immunoreactivity was reduced also in other structures related to stress responses, such as amygdala or cerebral cortex (Fig 7). Interestingly, S100A6 staining was detected in neurons somas and fibers in the amygdala of the control group (Fig 7C), whereas in the stressed group staining of the neurons somas was barely visible and it was absent in fibers (Fig 7F).

**Fig 8:**
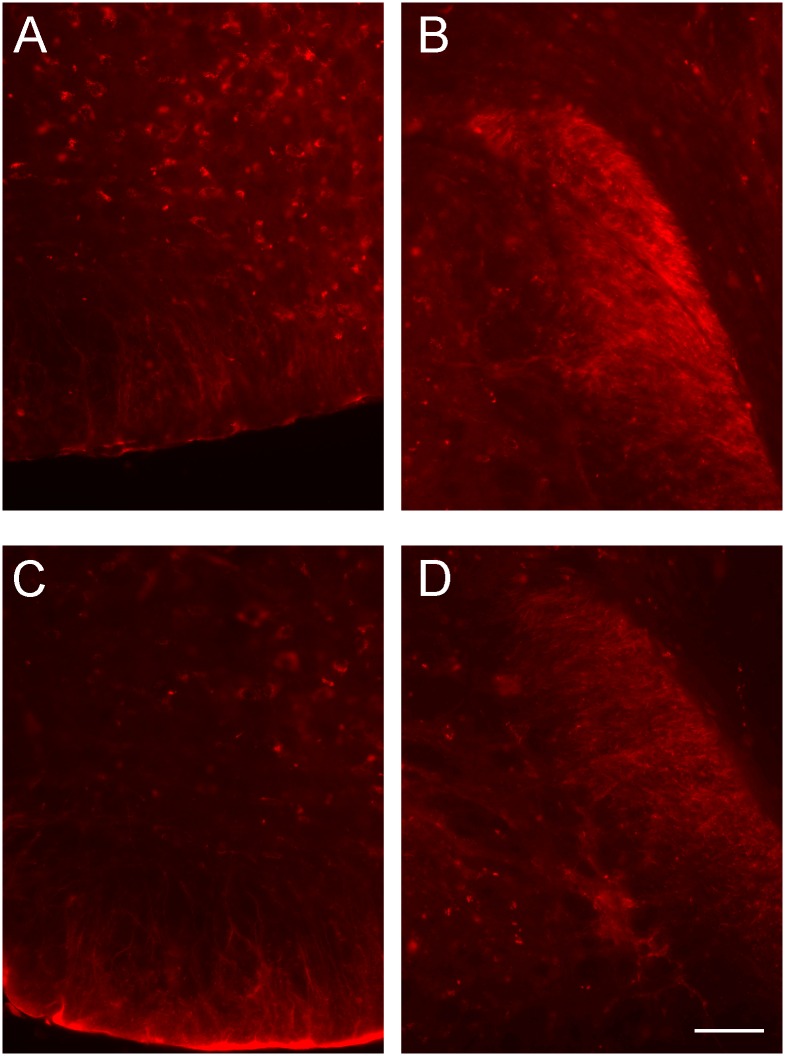
Representative immunofluorescence images for S100A6 in brainstem structures in control and stressed mice. (A, C) the pyramidal tract in control and stressed mice, (B, D) the spinal trigeminal tract. Scale bar equals 50 μ.

Cellular expression of S100A6 was detected within the brainstem, starting from midbrain to the medulla oblongata. Weak immunoreactivity of this protein was observed in serotoninergic, dopaminergic and noradrenergic neurons located in the brainstem, and prominent labeling was detected in main brainstem fiber tracts such as the pyramidal tract (Fig 8A and 8C) and also in fibers in some local nuclei, such as fibers within the A5 cell group. Numerous labeled S100A6-immonolabeled fibers were also found in the spinal trigeminal tract (Fig 8B and 8D). The pattern of S100A6 labeling in the brainstem structures was the same in control and stressed mice (Fig 8). The only difference between the two groups was in the intensity of immunofluorescence staining, which was weaker in the stressed group than in the controls (Fig 8).

## Discussion

We examined the involvement of S100A6 in stress responses, focusing our attention on the phenotype of cells expressing S100A6 in the mouse brain. Generally, the cells heavily labeled for S100A6 in all brain ventricle zones and in large fiber tracts such as the corpus callosum, external capsule or the superior cerebellar peduncles were astrocytes. Neurons expressing S100A6 at lower levels were located in the cerebral cortex, the hippocampus, the thalamus, the brainstem and the cerebellum. In the cerebral cortex cell bodies of neurons expressing S100A6 were densely placed in layers 2–3, absent in layer 4 and scarce in layers 5–6. Fibers labeled for S100A6 were visible in layers 2–6. The densest expression of S100A6 was seen in the visual and auditory visual areas, while labeling was barely visible in the somatosensory and motor areas. In the cerebral cortex of stressed animals, including the auditory cortex only lightly immunostained neurons’ somas were observed. In the stressed animals, the level of S100A6 protein in brainstem structures was one-third that in control mice. Decreased levels of this protein were also observed in the olfactory bulb, the SVZ of the lateral ventricles, the hippocampus, the hypothalamus and the amygdala.

### Changes in S100A6 levels in various brain structures in response to stress

S100A6 is present in many brain structures, being expressed in various brain cell types, including neurons and astrocytes, and is thought to have broad functions. S100A6 is linked to a variety of neurodegenerative diseases such as Alzheimer’s disease [10], amyotrophic lateral sclerosis, also called motoneuron disease [7], and disorders associated with aging [4]. In all these cases, as well as in normal aging, S100A6 is up-regulated. Increases in the levels of S100A6 mRNA have also been demonstrated in epilepsy [13, 14] and following traumatic brain injury [15]. Up-regulation of this protein has been observed in a rat model of epilepsy that is induced by amygdalar stimulation. It has been shown that in rats with induced epilepsy, the level of S100A6 protein was up-regulated in the cerebral cortex and the CA1 region of the hippocampus after 2 weeks of amygdalar stimulation. In the case of epilepsy and all of these other diseases, up-regulation of S100A6 was correlated with astrogliosis.

Although the S100A6 protein is usually up-regulated in neurodegenerative diseases, we found that in some brain structures, a decrease in S100A6 occurred in response to stress. In particular, in brainstem structures of stressed mice, the level of S100A6 was decreased to one-third that in control animals. The brainstem, consisting of the midbrain, pons and medulla oblongata, contains major fiber tracts and neuronal populations. Neurons present in the brainstem belong to the periaqueductal gray, reticular formation, red nucleus, serotoninergic, dopaminergic and noradrenergic nuclei and the cranial nerve nuclei. Thus, the brainstem controls many important and vital body functions including cardiac and respiratory function and coordination of motor control.

Some brainstem structures, particularly those belonging to the noradrenergic system (A1–A7), are considered to play crucial roles in response to stress [16]. A group of noradrenergic neurons (A1, A2 and some of the locus coeruleus neurons) sends massive inputs to the hypothalamus [17, 18]. These fibers form synapses on neurons producing corticotropin-releasing factor in the paraventricular nucleus of the hypothalamus. Increased activity of the noradrenergic locus coeruleus nuclei (and of the peripheral sympathetic nervous system, including the adrenal gland cortex) initiates the stress reaction and activates hypothalamic nuclei. To explain a decreased level of S100A6 in the brainstem in stress responses we determined cellular localization of this protein. Our data showed that S100A6 in the brainstem was mainly expressed in fibers. In A5, the somas of only a few neurons showed positive immunolabeling against S100A6, whereas fibers within this nucleus were strongly stained. Noradrenergic A5 neurons receive inputs from the paraventricular nucleus of the hypothalamus [19, 20]. Therefore, we suggest that S100A6 is involved in stress responses; in particular, it may be associated with homeostatic down-regulation of the hormone levels back to baseline. After changing environmental conditions, presenting stressors as a harmful event the sympathetic nervous system is activated and the fight-or-flight response is triggered. Once the harmful situation has passed the parasympathetic system then returns the organism to normal physiological conditions.

There is some evidence to indicate that different stressors activate different brain structures to elicit stress responses [21–23]. For example, the existence of stressor-specific pathways has been described for brainstem noradrenergic neurons, where immobilization stress activates noradrenergic brainstem neurons but metabolic stressors do not change their activation [24]. We used the unpredictable stress paradigm that lasts for 4 days. During that time, animals were exposed to severe stressors such as the forced swim test or predator stress. In addition to brainstem structures, the ARH also showed decreased intensity of S100A6 immunolabeling in stressed animals; thus, we believe that this protein is involved in adaptive responses to stress and might regulate HPA axis activity. The ARH is located in the mediobasal hypothalamus, close to the third ventricle, and consists of neuroendocrine neurons and projection neurons [25]. Immunohistochemical staining of the hypothalamus shows highly intense S100A6 labeling of the ARH. Although the ARH has cytoarchitectonic subdivisions, S100A6-immunopositive cells and mostly fibers are homogeneously distributed within this nucleus. Double staining immunohistochemistry revealed that a majority of S100A6 fibers in the ARH coincided with vimentin immunostaining indicating that S100A6 protein is present within tanycytes. Recently, it has been shown that tanycytes are involved in mediating neuroendocrine response to stress [26], which suggests that tanycytes in the ARH contribute to HPA axis regulation. On the other hand fibers that are confined within the hypothalamus and not expressed vimentin, most probably are unmyelinated axons and project to the anteroventral periventricular nucleus, medial preoptic nucleus and paraventricular nucleus, which are implicated in HPA axis adaptation to stress [27]. In turn, these hypothalamic nuclei send strong inputs to the ARC [28]. It has been shown that the ARH plays a crucial role in homeostasis, regulating food intake and body weight [29–31].

Reduction of the levels of S100A6 in the hypothalamus in response to stress suggests that this protein may be involved in reacting to the increased intracellular levels of the calcium ions that result from activity of the stress-related structures. It has been shown earlier that S100A6 interacts with several target proteins and after binding the calcium ion S100A6 is ubiquitinated and degraded [32], which may lead to temporal depletion of this protein. Interestingly, low levels of S100A6 protein and mRNA have been observed in rat models of traumatic brain injury [33], which is associated with reduced levels of glucocorticoid receptors in the hippocampus [34]. Our unpublished data also show that the level of glucocorticoid receptors in stress-related structures such as the hippocampus and the hypothalamus is reduced in the stressed mice.

### Can S100A6 protein be considered as a marker of adult neurogenesis?

In the current study, we provide the first comprehensive study of cells expressing S100A6 in response to stress and their localization in brain structures. Overall distribution of S100A6 in the brain of mouse is similar to that in the rat [3]. S100A6 is detectable in many brain structures, being expressed in various types of brain cells, including neurons and astrocytes. It has been shown that among numerous brain structures, S100A6 is also located in newly generated cells migrating along the rostral migratory stream to the olfactory bulbs. Yamada and Jinno [5] used several markers for proliferating cells to study the involvement of S100A6 in adult neurogenesis. They found that almost all S100A6-positive cells in the DG colocalized with proteins that mark newly generated cells. Based on these molecular characteristics of cells expressing S100A6 in the DG of the hippocampal formation, they proposed that S100A6 can be considered as a marker of neural stem cells.

The use of double labeling of the brain progenitor cells allowed us to verify this hypothesis. We examined the colocalization of S100A6 and SOX2, of which the latter is expressed in progenitor cells during development and adulthood, in two neurogenic structures: the DG of the hippocampal formation and the SVZ of the lateral ventricles. We found that the extent of colocalization of S100A6 and SOX2 differed between the DG and the SVZ of the lateral ventricles. The majority of SOX2-positive cells located in the DG simultaneously expressed S100A6, which is consistent with the study of Yamada and Jinno [5]. However, in the SVZ of the lateral ventricles, SOX2 expression was low. Only a minority of SOX2-expressing cells (approximately 30%) were also positive for S100A6. Thus, double immunofluorescence staining of sections for SOX2 and S100A6 showed that the latter protein could not be a reliable marker of progenitor cells, as only a small population of neural progenitor cells in the SVZ expressed S100A6.

In conclusion, our data show a highly differential expression of S100A6 in brain regions and within specific cell phenotypes. High levels of S100A6 in glial cells and tanycytes indicate that this protein plays a very special role in functioning of these cells, whereas low levels of S100A6 were detected in neurons of the hippocampus, the thalamus and other brain regions, including the cerebral cortex with greater immunostaining in the visual and auditory areas. The reasons for some details of this distribution (e.g. in various cortical areas) are not clear. On the other hand, reduction of the protein concentration after stress is a strong indication of its involvement in stress responses.
